# The College Museum

**Published:** 1867-10

**Authors:** 


***The College Museum.***

The beautiful rooms allotted to the museum have yet many unfilled spaces. Let our friends contribute specimens which they wish preserved, that they believe will be useful for teaching and illustration. Each will be carefully labelled and inscribed with the name of the donor or depositor. Mr. Tieman, the celebrated surgical instrument maker, of New York, has already deposited a full *suite* of instruments of his manufacture, and proposes to keep every department of his productions fully represented.

The Chicago College of Pharmacy, occupying spacious apartments in the building, exhibits one of the finest collections of materia medica and chemicals in this or any other country.

It is intended that the museum shall represent, *ad unguem*, the history and progress of medical science.

